# *In Vitro* Functional Analyses of Arrhythmogenic Right Ventricular Cardiomyopathy-Associated Desmoglein-2-Missense Variations

**DOI:** 10.1371/journal.pone.0047097

**Published:** 2012-10-10

**Authors:** Anna Gaertner, Baerbel Klauke, Ines Stork, Karsten Niehaus, Gesa Niemann, Jan Gummert, Hendrik Milting

**Affiliations:** 1 Erich and Hanna Klessmann Institute for Cardiovascular Research and Development, Clinic of Thoracic and Cardiovascular Surgery, Heart and Diabetes Centre NRW, Ruhr-University Bochum, Bad Oeynhausen, Germany; 2 Department for Proteome and Metabolome Research, Faculty of Biology and Centre for Biotechnology-CeBiTec, Bielefeld University, Bielefeld, Germany; 3 Organic and Bioorganic Chemistry, Bielefeld University, Bielefeld, Germany; Northwestern University, United States of America

## Abstract

**Background:**

Although numerous sequence variants in desmoglein-2 (DSG2) have been associated with arrhythmogenic right ventricular cardiomyopathy (ARVC), the functional impact of new sequence variations is difficult to estimate.

**Methodology/Principal Findings:**

To test the functional consequences of DSG2-variants, we established an expression system for the extracellular domain and the full-length DSG2 using the human cell line HT1080. We established new tools to investigate ARVC-associated DSG2 variations and compared wild-type proteins and proteins with one of the five selected variations (DSG2-p.R46Q, -p.D154E, -p.D187G, -p.K294E, -p.V392I) with respect to prodomain cleavage, adhesion properties and cellular localisation.

**Conclusions/Significance:**

The ARVC-associated DSG2-p.R46Q variation was predicted to be *probably damaging* by bioinformatics tools and to concern a conserved proprotein convertase cleavage site. In this study an impaired prodomain cleavage and an influence on the DSG2-properties could be demonstrated for the R46Q-variant leading to the classification of the variant as a potential gain-of-function mutant. In contrast, the variants DSG2-p.K294E and -p.V392I, which have an arguable impact on ARVC pathogenesis and are predicted to be *benign,* did not show functional differences to the wild-type protein in our study. Notably, the variants DSG2-p.D154E and -p.D187G, which were predicted to be *damaging* by bioinformatics tools, had no detectable effects on the DSG2 protein properties in our study.

## Introduction

Arrhythmogenic right ventricular cardiomyopathy (ARVC) is a heart muscle disease, characterised by non-ischemic ventricular arrhythmias and progressive dystrophy of the myocardium with fibrofatty replacement (reviewed in [1]). ARVC is a leading cause of exercise-related sudden death in people <35 years old [2], with an estimated prevalence of 6∶10,000 to 44∶10,000 [3]. Although the initial reports of ARVC described the right ventricular disease, left ventricular involvement or even global dilated cardiomyopathy also occur [4]. The occurrence of biventricular disease is also supported by genomics data, which have revealed that only a small number of genes are differentially regulated between the right and left ventricle in ARVC [5]. Myocyte loss accompanied by inflammation, fibrosis and adiposis mainly within the right ventricular wall are the histological hallmarks of the disease [6]–[8].

Between 50% and 80% of ARVC cases are regarded as familial and have autosomal dominant inheritance in most cases [1], [6], [9]. Estimating the amount of familial cases remains difficult due to reduced and age-dependent penetrance combined with variable and incomplete disease expression within a family [10], [11].

According to the National Center of Biotechnology Information (NCBI) database at least 12 chromosomal loci for ARVC and some of the causal genes have been identified, reflecting high genetic heterogeneity. Five genes (*DSP*, *PKP2*, *DSG2*, *DSC2* and *JUP*) code for the desmosomal proteins desmoplakin, plakophilin-2, desmoglein-2, desmocollin-2 and plakoglobin, respectively [9], [10], [12]–[15].

Desmosomes are highly organised anchoring junctions that are primarily abundant in tissues that are subject to frictional and shear stress (reviewed in [16]). As *maculae adhaerentes* they are intercellular adhering junctions which secure the structural and functional integrity of cardiac tissue [17]–[19]. Together with adherens junctions (*fasciae adhaerentes*) and gap junctions, they localise to the intercalated discs (ID), the main site of myocyte interconnection [17], [20]. However, colocalisation of the usually distinct components of desmosomes and adherens junctions is observed in a complex and intimate structure termed *area composita* in the human ID [17].

In desmosomes, the single-pass type I transmembrane glycoproteins of the cadherin family, the desmocollins (DSCs) and the desmogleins (DSGs), interact to form the adhering interface [21], [22]. Via their extracellular cadherin domains EC1-4 they can interact either in a homophilic or heterophilic Ca^2+^-dependent manner [22]–[25]. The Ca^2+^-dependent cell adhesion molecules are synthesised as precursor polypeptides [26]. As shown previously human DSG1 and DSG3 precursors are efficiently maturated by the cleavage of their prodomain through proprotein convertase (PC) furin activity [27]. Of the four known desmoglein isoforms DSG2 is believed to be the only one present in cardiac tissue [28]–[31].

Although between 30% and 40% of ARVC cases harbour sequence variants in one of the known disease-causing genes [11] the etiopathogenesis of the disease remains poorly understood. Rather than containing mutational hot spots, *DSG2* is mutated in numerous and varied locations spread over the complete open reading frame. Therefore, interpretation of missense mutations/variations for genetic counselling still remains challenging [11].

In this study, we recombinantly expressed the extracellular cadherin domains (EC1-4) of wild-type and mutant DSG2 (rECD) and subsequently analysed their secondary structure and prodomain cleavage. We further tested the homo-dimerisation properties of these molecules in solution and their cellular adhesion properties with a flow cytometry-based binding assay. Additionally, we examined the cellular localisation of *full-length* DSG2-EYFP (fl-DSG2-EYFP) chimeras in cells. For the analysis, we selected five ARVC-related DSG2-variants located within the extracellular domain (ECD; Table 1): p.R46Q [12], p.D154E [32], p.D187G [33], p.K294E [34], p.V392I [35]. Our goal was to analyse the functional impact of sequence variations that have been reported to be associated with ARVC.

**Table 1:** Investigated DSG2-variants.

variant labelling	R46Q	D154E	D187G	K294E	V392I
**protein change**	p.Arg46Gln	p.Asp154Glu	p.Asp187Gly	p.Lys294Glu	p.Val392Ile
**DNA change**	c.137G>A	c.462C>A	c.560A>G	c.880A>G	c.1174G>A
**domain**	prodomain	EC1	EC2	EC3	EC4
**number of clinical reports**	7	2	1	2	11
**prevalence in controls**	0/1120	0/400	0/1400	0/560	2/3530^a^
**patient classification**	TFC+	TFC+	-	TFC+	TFC+, DCM
**Grantham Score** b	43	45	94	56	29
**SIFT** c	affect protein function	affect protein function	affect protein function	affect protein function	tolerated
**PolyPhen** d	probably damaging	possibly damaging	probably damaging	benign	benign
**cosegregation within family**	only in a few clinical reportsfamily members availablefor genetic testing; insome families incompletecosegregation	cosegregation within family: two additional affected variant carriers	no family membersavailable for genetictesting	no family membersavailable for genetictesting	incomplete cosegregation
**notes**	in one patient DSG2-p.Arg46Gln digenic with a PKP2-variant; two individuals presented with the nonsynonymous SNP DSG2-p.Val158Gly on the same allele as DSG2-p.Arg46Gln	-	-	-	in some cases DSG2-p.V392I occurs digenic with PKP2- or DSC2-variants

All data were obtained from the ARVC database [64] and the corresponding references. TFC = task force criteria, EC = extracellular cadherin domain, DCM = dilatative cardiomyopathy.

a The prevalence in controls for the DSG2-V392I was adapted to the results in our research group [35].

b Grantham, R. (1974). “Amino acid difference formula to help explain protein evolution.” Science 185(4154):862–864.; Li, W. H., C. I. Wu, et al. (1984). “Nonrandomness of point mutation as reflected in nucleotide substitutions in pseudogenes and its evolutionary implications.” J Mol Evol 21(1): 58–71.

c Kumar, P., S. Henikoff, et al. (2009). “Predicting the effects of coding non-synonymous variants on protein function using the SIFT algorithm.” Nat Protoc 4(7): 1073–1081.

d Ramensky, V., P. Bork, et al. (2002). “Human non-synonymous SNPs: server and survey.” Nucleic Acids Res 30(17): 3894–3900.

## Results

### Wild-type and Variant DSG2-EC1-4 Proteins can be Expressed in HT1080 Cells

**Figure 1:**
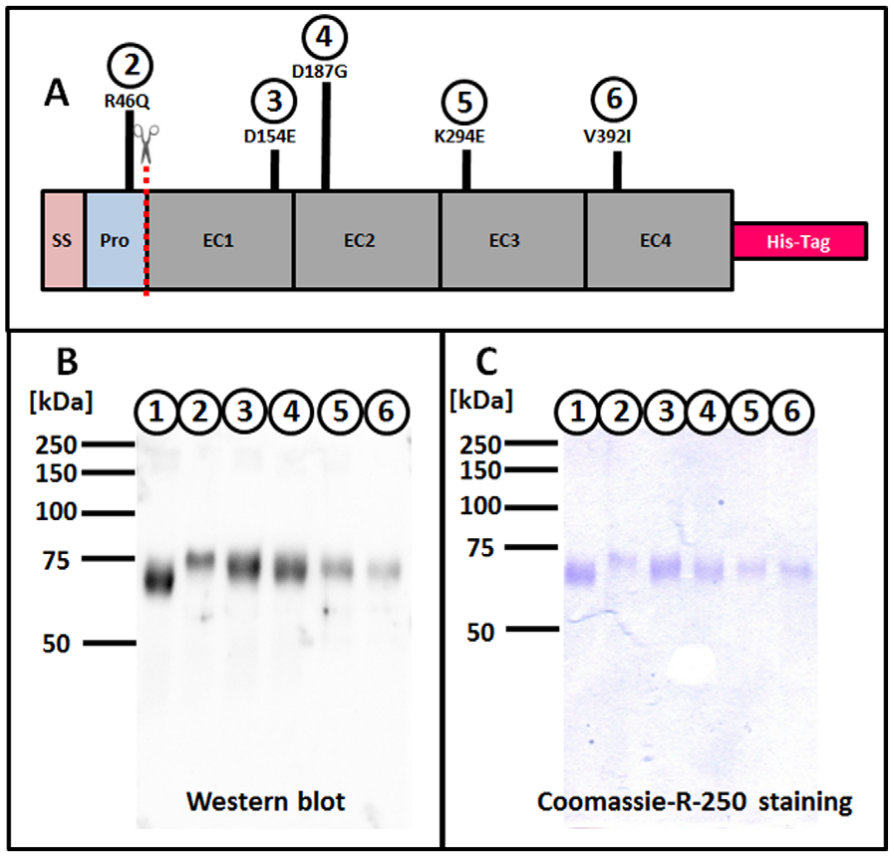
A **Schematic view of the rECD with analysed ARVC-associated variations.** The dotted line shows the predicted PC cleavage site. SS = signal sequence, Pro = prodomain, EC1-EC4 = DSG2 extracellular cadherin subdomains 1-4. **B** Recombinantly expressed proteins were identified as DSG2-ECD with anti-DSG2-10G11 by Western blot analysis. The calculated apparent molecular weights were 67.5±1.5, 72.5±3.5, 70.0±3.0, 70.0±3.0, 70.5±2.5, and 69.0±4.0 (mean±SEM; n = 2) for the proteins in the traces in 1, 2, 3, 4, 5 and 6, respectively. **C** Coomassie-R-250 staining revealed the purity of the proteins. 1 = rECD-wt, 2-6 = rECDs as labelled in **A**.

DSG2-EC1-4 was recombinantly expressed in human fibrosarcoma HT1080 cells as wild-type (rECD-wt) and variants p.R46Q (rECD-R46Q), p.D154E (rECD-D154E), p.D187G (rECD-D187G), p.K294E (rECD-K294E), p.V392I (rECD-V392I) (Figure 1A). rECD analysis with SDS-PAGE and subsequent Coomassie-R-250 staining, Western blot and Matrix assisted laser desorption/ionization-peptide mass fingerprint (MALDI-PMF; data not shown) revealed that the recombinantly expressed proteins were characterised as DSG2-ECD (Figure 1B)- with a molecular weight of approximately 70 kDa and a purity of >90% (Figure 1C).

Note that, although the variants differ from the wild-type protein only by one amino acid, the migration of rECD-variants in SDS-PAGE differed from that of the rECD-wt. In the case of rECD-R46Q, differing electrophoretic velocities could be explained by altered processing with MALDI-in-source decay (MALDI-ISD). For the other variants, the deviating mobility might result from different glycosylation patterns as compared to the wild-type molecule.

### rECD-wt Secondary Structure, Consisting Mainly of β-strands, is Affected by Ca^2+^

**Figure 2:**
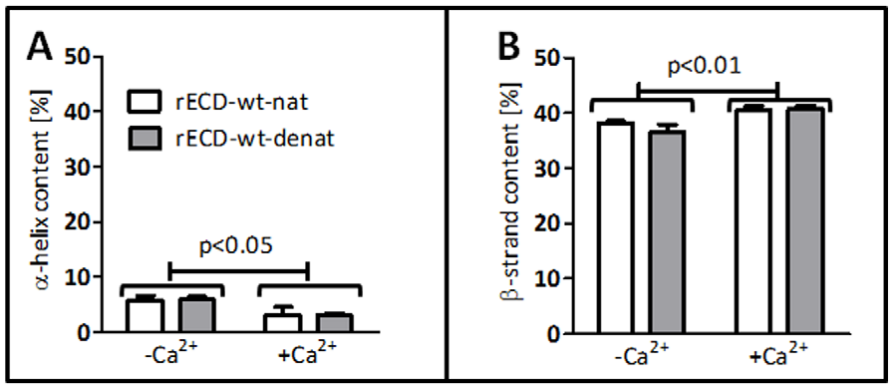
Evaluation of CD data. Results of the deconvolution with DichroWeb [67] using the CONTINLL-algorithm [68], [69] and the CRYST175 [70] reference data set. The results are presented as means±SEM [%] for three independent measurements. α-helical content (**A**) was 5.9±0.8 and 5.9±0.5 without Ca^2+^ (-Ca^2+^) and 3.2±1.4 and 3.2±0.2 with 5 mM CaCl_2_ for rECD-wt-nat and rECD-wt-denat, respectively. β-strand content (**B**) was 38.2±0.3 and 36.6±1.3 without Ca^2+^ and 40.5±0.8 and 40.7±0.6 with 5 mM CaCl_2_. Analysis of the secondary structure with two-way ANOVA showed that the α-helix content (**A**) was significantly decreased (p<0.05) while the β-strand content (**B**) was significantly increased (p<0.01) by the addition of 5 mM CaCl_2_ (+Ca^2+^). However, as shown by two-way ANOVA, purification conditions had no significant effect on the rECD secondary structure.

Since at least one of the protein variants (rECD-V392I) could not be purified under native conditions by affinity chromatography (data not shown), we purified all rECDs under denaturating conditions for a better comparability. As a control we evaluated the influence of the purification conditions on the secondary structure of rECD-wt by circular dichroism (CD). We did not find evidence for structural differences between denaturating and native conditions by CD-spectroscopy. Evaluation of CD data with DichroWeb (http://dichroweb.cryst.bbk.ac.uk/) revealed that the recombinant proteins mainly consisted of β-strands with only a small ratio of α-helices (Figure 2).

The addition of 5 mM Ca^2+^ led to a significant decrease of α-helical and to a significant increase of β-strand content (Figure 2), indicating the dependency of the secondary structure of the rECD-wt on the presence of Ca^2+^. Additional CD data (molar circular dichroism; Δε) are presented in the Supporting Information (Figure S1).

### ARVC-associated DSG2-p.R46Q Inhibits the Prodomain Cleavage of rECD

Prodomain cleavage was previously shown to be essential for the maturation of other DSG isoforms [27]. A consensus PC cleavage site in DSG2 is located at amino acids 46-49 (positions 1-4 of the cleavage site sequence logos; Figure S2). In the DSG2-p.R46Q variant, the arginine at position 1 is substituted by a glutamine, thereby eliminating the potential PC cleavage site.

**Figure 3:**
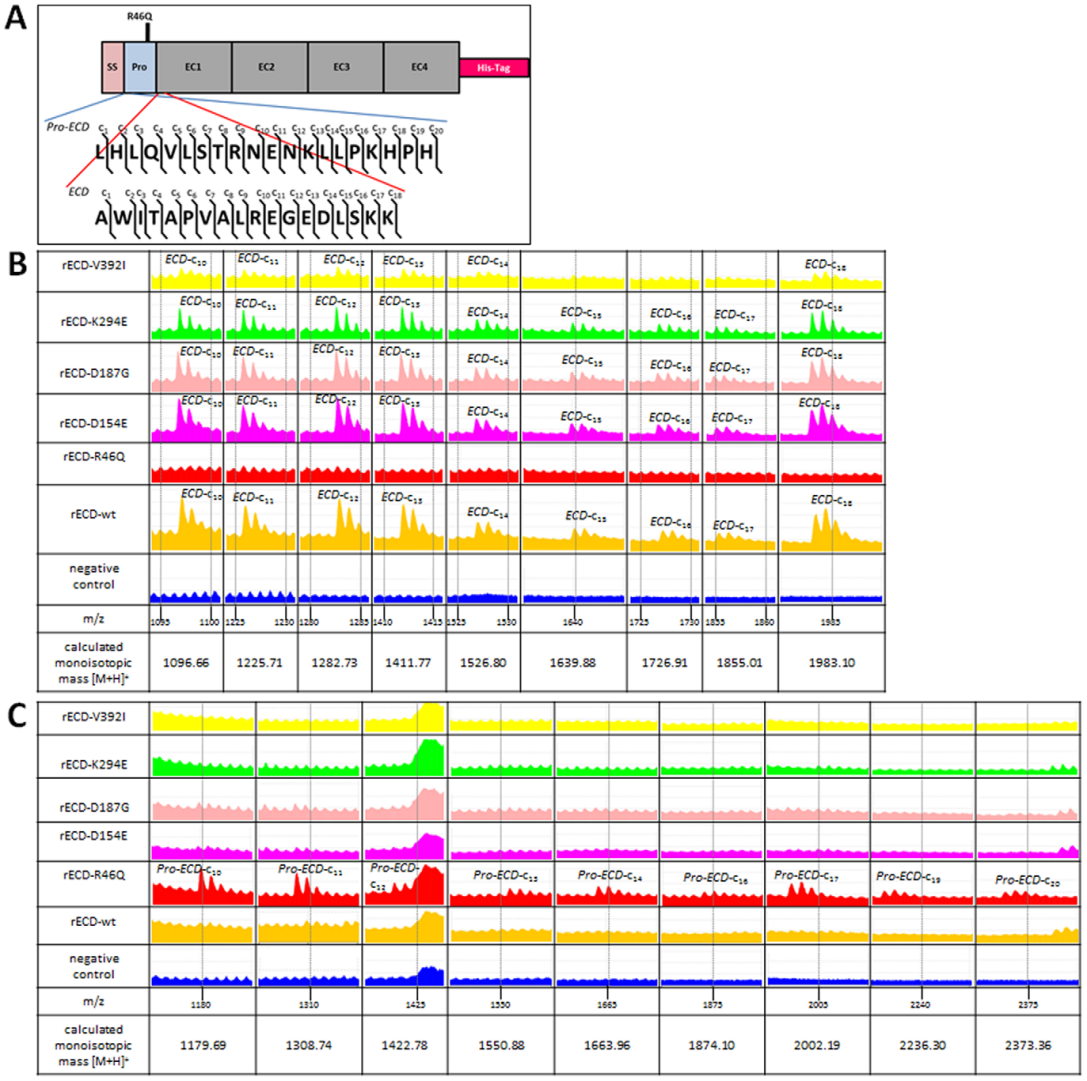
Comparison of rECD fragment ion peaks generated by MALDI-ISD using QuPE [**71**]. **A** The location of *ECD* and *Pro-ECD* and the positions of the corresponding c-ions are illustrated on the schematic view of the rECD. **B+C** Each row shows extracts of the MALDI-ISD spectra for the particular rECDs. Peaks in one column represent the ions of the m/z ratios indicated below the negative control. The corresponding c-ions are indicated for each peak. The calculated monoisotopic masses [M+H]^+^ are shown at the bottom of each column. **B** Fragment ion peaks representing the *ECD*-c-ions; only rECD-R46Q shows no fragment ions corresponding to *ECD* **C** Fragment ion peaks representing the *Pro-ECD-*c-ions; only rECD-R46Q shows fragment ions corresponding to *Pro-ECD*.

Amino acid sequences of the N-terminus of the rECDs and prodomain cleavage were examined by MALDI-ISD. N-terminal fragmentation corresponding to *SS-Pro-ECD-*c-ions (DSG2-preproprotein) was not observed for any of the rECDs (data not shown), demonstrating proper signal sequence cleavage. Likewise, the prodomain was properly cleaved for all rECDs with the exception of rECD-R46Q, as demonstrated by the peaks corresponding to *ECD*-c-ions (matured DSG2) that were detected (Figure 3B). The lack of *ECD-*c-ions and the presence of *Pro-ECD*-c-ions (DSG2-proprotein) for rECD-R46Q (Figure 3C) signify that cleavage of the rECD-R46Q-prodomain failed.

### rECD-R46Q Shows an Increased Binding to HT1080 Cells as Compared to rECD Wild-Type and Other Variants in the Flow Cytometry-based Adhesion Assay

**Figure 4:**
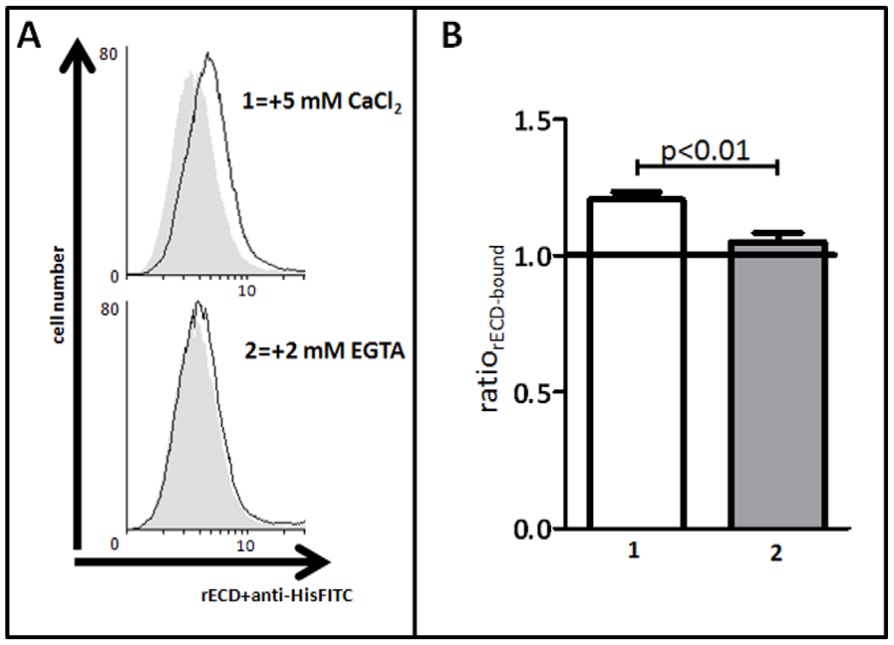
Flow cytometry-based assay for rECD-binding. **A** Representative histograms of FITC-fluorescence for rECD-wt binding with 5 mM CaCl_2_ (1) or with 2 mM EGTA (2). HT1080 cells were incubated with (black line) or without (negative control, grey filled area) rECD-wt. Bound rECD-wt was detected with anti-HisFITC. **B** Column plots representing the ratios of rECD-wt-binding (ratio_rECD-bound_; for calculation see Supporting Information) indicated as mean±SEM of 3 independent measurements as detected by flow cytometry. The ratio_rECD-bound_ was significantly (p<0.01) decreased from 1.21±0.03 for samples incubated in 5 mM CaCl_2_ (1) to 1.05±0.03 for samples incubated with 2 mM EGTA (2) showing that rECD-wt has a Ca^2+^-dependent binding to HT1080 cells. Statistical analysis was performed with unpaired student’s t-test (GraphPad Prism 5.01).

The human fibrosarcoma cell line HT1080 [36], used here as a model system for the flow cytometry-based adhesion assay, is similar to the human cardiac *area composita* of the ID [17], [37] in that it expresses the desmosomal cadherins DSG2 and DSC2 [22], [38] as well as the classical cadherin N-cadherin (Figure S3). To establish the cellular adhesion assay, the binding of 0.8 µM rECD-wt to HT1080 was analysed in 5 mM CaCl_2_ or in 2 mM EGTA (Figure 4). rECD-wt-binding to HT1080 was detectable in samples incubated with 5 mM CaCl_2_, whereas EGTA led to a significant (p<0.01) decrease of flow cytometry-detectable rECD-binding. Further information about how the Ca^2+^-dependent cell adhesion model was established is available in the online Supporting Information (Figure S4 and S5).

**Figure 5:**
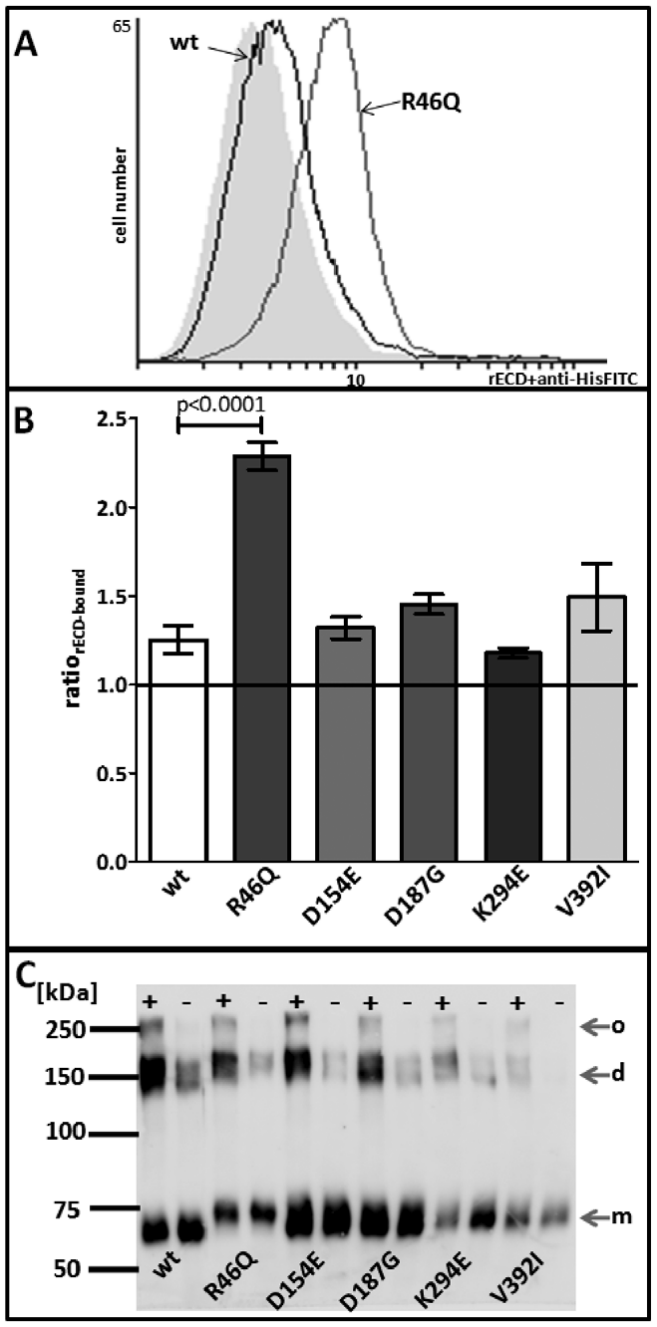
Adhesion properties of rECDs. **A+B** Flow cytometry-based assay for the binding of 0.8 µM rECD-wt or -variants to HT1080. **A** Representative histograms of FITC- fluorescence for binding of rECD-wt- and rECD-R46Q (as indicated). Bound rECD was detected with anti-HisFITC. As a negative control, HT1080 cells were incubated with only anti-HisFITC (grey filled area). **B** Column plots representing the ratio of rECD-binding related to the negative control (ratio_rECD-bound_) as detected by flow cytometry. Ratios_rECD-bound_ are indicated as mean± SEM of 7 independent measurements for rECD-variants and 9 independent measurements for rECD-wt with rECDs from at least 3 different purifications. Statistical analysis was performed by one-way ANOVA with Dunnett’s posttest using rECD-wt as a control (GraphPad Prism 5.01). rECD-R46Q-binding to HT1080 is increased 1.8-fold as compared to rECD-wt. Other ARVC-associated variants have no influence on rECD-binding to HT1080. **C** Representative Western blot (with anti-DSG2-10G11) of rECDs crosslinked in a 5 mM CaCl_2_ containing buffer with BS^3^ (+) or of controls (-) reveals that rECD wild-type and variants exist in solution as monomers (m), dimers (d), and oligomers (o).

Statistical analyses on the ratio of rECD-binding as compared to that of controls revealed that no rECDs except for rECD-R46Q showed significant difference in binding to HT1080 (Figure 5A, 5B). In contrast to the others, the ratio of rECD-R46Q-binding to HT1080 increased significantly (2.29±0.08) as compared to the rECD-wt (1.25±0.08; p<0.0001).

We assumed that rECDs bind to DSG2 expressed by HT1080 cells. Although siRNA knock-down led to a reduction of available DSG2 epitopes (Figure S6), we could not detect a reduced binding of rECD-wt after DSG2 knock-down (Figure S7). The identity of further rECD-binding partners on HT1080 remained unclear.

### ARVC-associated DSG2 Variations do not Influence Homo-oligomerisation Properties of rECD

Protein interactions can be identified by crosslinking neighbouring proteins by suberic acid bis(3-sulfo-N-hydroxysuccinimide ester) sodium salt (BS^3^) and subsequent SDS-PAGE. BS^3^ treatment of rECDs in 5 mM CaCl_2_ resulted in the formation of rECD dimers and oligomers (Figure 5C). Reproducibility of rECD oligomer formation was verified for two independent rECD purifications. Since dimerisation and oligomerisation of rECDs were found for both the wild-type and the rECD-variants, we conclude that the investigated ARVC-related DSG2-variants have basically no influence on the multimerisation properties of rECD in solution (Figure 5C).

### The Cellular Localisation of fl-DSG2-EYFP with ARVC-associated Variations is Comparable to that of the Wild-type

**Figure 6:**
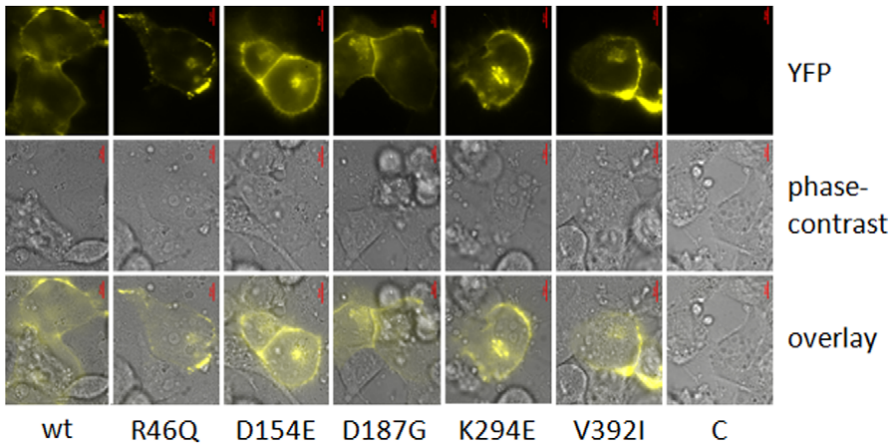
Representative images for detecting the localisation of wild-type and variants of full-length-DSG2-EYFP in HT1080 for three independent transfection experiments: **DSC2b-HT1080 cells were transfected with full-length(fl)-DSG2-pEYFP; live cells were analysed with a fluorescence miscroscope one day after transfection.** R46Q, D154E, D187G, K294E and V392I indicate the sequence variant in fl-DSG2-EYFP, wt fl-DSG2-wt-pEYFP, and C the LFA mock transfected control. Chimeric DSG2-proteins localised preferentially to the cell borders. ARVC-associated variations had no detectable influence on the localisation of fl-DSG2-EYFP in DSC2b-HT1080. Images were acquired through YFP and phase-contrast filters. Scale (red bar) = 10 µm.

fl-DSG2-wt-EYFP, -R46Q-EYFP, -D154E-EYFP, -D187G-EYFP, -K294E-EYFP and -V392I-EYFP were expressed in HT1080 cells stably transfected with DSC2b (DSC2b-HT1080; for characterization, see Figure S8). Fluorescence microscopy of transiently transfected live cells revealed a predominant localisation of the chimeric full-length-DSG2-proteins at the cell borders (Figure 6) and clusters in proximity of the nucleus (data not shown). ARVC-associated variations had no detectable influence on the localisation of fl-DSG2-EYFP in DSC2b-HT1080 cells. A shift towards increased intracellular localisation was not observed.

## Discussion

Since the initial characterisation of *DSG2* as an ARVC-associated gene in 2006 further disease-linked variants have been identified in the desmosomal cadherins [12], [32]–[35], [39]. As clinical diagnosis in ARVC is challenging, genetic screening plays an increasing role in the clinical classification of familial forms of ARVC. In the revised Task Force Criteria (TFC) for classic right-dominant subtype of ARVC, *Identification of a pathogenic mutation categorized as associated or probably associated with ARVC/D in the patient under evaluation* is even one of the major criteria [40]. But the high prevalence of “private” mutations in ARVC patients makes interpreting genetic test results highly challenging, since the pathogenic influence of each novel variation has to be confirmed. According to TFC a pathogenic mutation is defined as a *DNA alteration associated with ARVC/D that alters or is expected to alter the encoded protein, is unobserved or rare in a large, non-ARVC/D control population, and either alters or is predicted to alter the structure or function of the protein or has demonstrated linkage to the disease phenotype in a conclusive pedigree* [40]. A transgenic mouse model only exists for one gene variation (DSG2-p.N266S) so far [41], and molecular pathomechanisms in desmosomal cadherin variants have been investigated in only a few studies [42]–[45]. Important goals of our study were therefore to develop tools that can be directly applied to novel missense variations in DSG2-ECD, and to gain further insight into the functional consequences of sequence variations for the development of ARVC.

We chose five ARVC-associated DSG2-variations localised in different extracellular subdomains of DSG2 that were previously published by us and others [12], [32]–[35] (Table 1). The DSG2-p.R46Q mutation [12] affects a conserved PC recognition motif. Variations DSG2-p.D154E [32] and DSG2-p.D187G [33] are localised within the conserved Ca^2+^ binding sites (Figure S9). In contrast, the two other variations, DSG2-p.K294E [34] and DSG2-p.V392I [35], are located in the extracellular cadherin domains 3 and 4, respectively.

The extracellular domain of desmogleins has been previously used as an independent functional unit, e.g. for nanomechanical interaction analysis [25], [46], [47]. We therefore consider the recombinantly expressed EC1-4 of DSG2 (rECD) to be an appropriate model for the biochemical studies of ARVC-associated DSG2-variants. The recombinantly expressed rECD-wt was confirmed by CD to consist mainly of β-strands. This is in agreement with the data obtained by Sano et al. (PDB ID 2YQG [48]), who found 4% helical and 34% β-sheet content for the first extracellular domain of DSG2. Since our data on the secondary structures of the DSG2-EC1-4 were comparable (Figure 2), we concluded that the purified molecules were appropriate for subsequent experiments.

It is well known that Ca^2+^ binds to desmosomal cadherins and induces conformational changes in the ECD, as has been previously shown by CD for the first two extracellular domains of DSG2 and DSC2 [24]. Of note, we show here for the first time that the addition of Ca^2+^ indeed leads to an increase of the β-strand content in the extracellular cadherin domains EC1-4. However, the rECD consists mainly of β-strands even under Ca^2+^-free conditions, which indicates that the basic structure of the extracellular domain is not dependent on the presence of Ca^2+^. Rather Ca^2+^ just induces further β-strand formation, presumably in proximity to the Ca^2+^ coordination sites. These changes might also induce an alteration of the tertiary structure that has been detected for classical cadherins upon Ca^2+^-binding [49], [50].

We should also mention that, although rECD-V392I could not be purified under native conditions, deconvolution of CD data on this variant showed no significant deviation of α-helix and β-strand content as compared to the wild-type protein (data not shown).

Cadherins, including the desmosomal cadherins, are synthesised as inactive precursors, which are post-translationally processed by removal of the N-terminal prodomain. In a baculovirus overexpression system DSG1 and DSG3 were shown to be substrates for the PC furin [27]. The wild-type DSG2 precursor contains a dibasic recognition motif (RQKR) predicted to be appropriate for cleavage by furin and other PCs active in the early secretory pathway such as PACE4, PC5/6 and PC7/8 [51]–[54]. By analysing the N-terminal sequence of the recombinantly expressed extracellular domain of DSG2 with MALDI-ISD we revealed that the sequence corresponded to the mature DSG2. This proves that the signal sequence and the prodomain were cleaved from the DSG2 molecule in our HT1080 cell model system. However, for the DSG2-p.R46Q-variant, MALDI-ISD analysis of the N-terminus showed that the molecule still carried the prodomain but lacked the signal sequence. These findings support the assumption that DSG2 like DSG1 and DSG3 is matured by a PC, which specifically recognises a dibasic motif. Additionally, it is obvious that the signal sequence is cleaved independently and earlier during intracellular maturation, and that it is located after amino acid 23, in agreement with the cleavage site predicted by SignalP 4.0 [55] and the UniProtKB/Swiss-Prot database [56] (accession number Q14126).

The presence of the prodomain in the rECD-R46Q-variant explains its deviating electrophoretic mobility as compared to the wild type protein (Figure 1). For the other investigated variants it is speculated that the deviating electrophoretic mobilities might result from different glycosylation patterns between wild type and variants. The approval of this assumption is in the focus of future studies.

For the classical cadherins, maturation by prodomain cleavage has been predicted to be necessary for the adhesive functions of the molecules [26], [57]. Only classical (type I) cadherins and desmocollins have prodomains of considerable length. It has been confirmed that the prodomain of murine N-cadherin (134 aa) contains a cadherin-like fold without homophilic interaction capabilities [57]. Since even an incomplete prodomain cleavage results in loss of the adhesion properties of E-cadherin [26] we predicted that even the comparably small prodomain of DSG2 (26 aa) would influence the adhesion of the p.R46Q-variant. Surprisingly, compared to the wild-type the mutant molecule showed an increase in binding of about two-fold; this could be due to yet unknown binding properties of DSG2’s prodomain. Aside from the p.R46Q variant, no other rECDs investigated differed in their adhesion properties to HT1080 from the wild-type molecule under the investigated conditions. The reason for the increased cellular adhesion of the p.R46Q variant was not investigated in this study but should be analysed in future studies, for example by using nanomechanical measurements. Nevertheless, we postulate that the p.R46Q variant is a gain-of-function mutation, since it *confers new or enhanced activity* to the protein.

The flow cytometry-based assays were performed under unphysiologically high Ca^2+^-concentrations (5 mM CaCl_2_). However, this Ca^2+^-concentration was also previously used for the analysis of the first two extracellular subdomains of DSG2 [24]. Since two of the variants (p.D154E and p.D187G) are predicted to affect the Ca^2+^-binding regions (Figure S9) the molecules might be sensitive to moderate Ca^2+^-changes.

We further analysed the rECD binding properties by crosslinking experiments and found that the rECDs of all variants and the wild-type formed homo-oligomers.

The human fibrosarcoma cell line HT1080 [36] can be regarded as an appropriate cell adhesion model for the study of ARVC-associated DSG2-variants. On the one hand HT1080 cells are convenient for the development of a robust and simple assay to investigate the binding properties of the extracellular domains of DSG2 to the surface of human cells. This is important since FACS analysis of cardiomyocytes is tricky, and human cardiomyocytes are difficult to handle and obtain. On the other hand HT1080 cells are comparable to cardiomyocytes [17], [37] as they express the classical cadherin N-cadherin (Figure S3) as well as the desmosomal cadherins DSC2 and DSG2 (Figure S3; [38]). This is important considering the fact that siRNA knock-down of DSG2 on HT1080 did not lead to reduced binding of rECD-wt in our study. As heterophilic interactions between DSG and DSC have been previously shown [22], [24], the rECD might therefore also bind to the DSC2 or even to N-cadherin present on the surface of HT1080 cells in the DSG2 knock-down experiments.

Since the synthesis of DSG2 needs several posttranslational modifications such as glycosylation and vesicular trafficking (from the ER to the Golgi apparatus, and finally to the plasma membrane), ARVC-related variants might affect transport or modification of the molecule. As a type I transmembrane protein [21], [58] the DSG2-ECD should be cotranslationally translocated into the ER. Within the ER, a strict quality control (QC) hinders incompletely folded proteins from leaving the ER and entering the cell-surface where they could be potentially damaging [59], [60]. Chimeras of a desmosomal and a fluorescent protein were previously used to analyse the localisation and behaviour of desmosomal proteins within cells [42], [61], [62]. We used chimeras of fl-DSG2 C-terminally coupled to EYFP to analyse the localisation of wild-type and mutant molecules in living HT1080 cells. Since all chimeras were transferred to the plasma membrane, we concluded that the missense variations do not have a major impact on intracellular QC. Similarly, no influence of intracellular ARVC-associated DSG2-variations on the localisation of fl-DSG2-EGFP chimeras was shown by Gehmlich et al. in primary neonatal rat cardiomyocytes [42]. In contrast to these results we (data not shown) and others [44], [63] have found that some DSC2-fluorescent protein chimeras carrying other ARVC associated variations (DSC2-p.R203C, -p.I231T, -p.T275M, -p.D350Y, -p.A897KfsX4) were mislocalised as compared to the wild-type protein.

While we can assume that rECD-R46Q is not properly processed, it is interesting to note that fl-DSG2-R46Q-EYFP appeared to be transported correctly to the plasma membrane. This is comparable to mutant E-cadherin [26] and N-cadherin [57] which are also fully integrated into the plasma membrane when expressed together with their prodomains.

In summary, we present here an expression system for analysing the extracellular domain and full-length human DSG2. To our surprise we could not find relevant differences *in vitro* in the binding properties or the cellular localisation, respectively, for the variants DSG2-p.D154E and -p.D187G.

According to Table 1, these variants were classified by a predictive algorithm as *possibly* or *probably damaging*. In contrast the variants p.K294E and p.V392I were classified *benign*. Furthermore with respect to the clinical data the pathogenic impact of these variants remains debatable. Of note, DSG2-p.R46Q, which was classified as *probably damaging* [64], was properly transported to the plasma membrane despite its aberrant post-translational processing. However, flow cytometry analysis suggested that this variant is a gain-of-function rather than a loss-of-function mutation.

## Materials and Methods

### Cloning and Mutagenesis of DSG2 and DSC2b cDNA Constructs

Human full-length (fl) DSG2 and DSC2b cDNA were obtained by reverse transcription of RNA isolated from explanted cardiac tissue. The myocardial samples were obtained from patients undergoing cardiac transplantation and prepared according to standardised operating procedures (SOP). The investigation conforms to the principles outlined in the Declaration of Helsinki. The study was approved by the ethics committee of the Ruhr-University Bochum in Bad Oeynhausen, and all patients provided written informed consent for the use of tissue samples.

To generate plasmids coding for DSG2-EC1-4-Arg-Gly-Ser(RGS)-6xHis, fl-DSG2 and fl-DSC2b cDNA were amplified with appropriate primers (Table S1) and subcloned into pLPCX (Clontech) or pEYFP-N1 (Clontech). The ARVC-associated variations (DSG2-p.R46Q, -p.D154E, -p.D187G, -p.K294E, -p.V392I) were introduced either by site-directed mutagenesis using the QuikChange® Lightning Site-Directed Mutagenesis Kit (Stratagene), or using cDNA with the required variation. DSG2-EC1-4-RGS-6xHis-pLPCX plasmids were used for the recombinant expression of DSG2-EC1-4 (rECD), DSG2-pEYFP-N1-plasmids for fl-DSG2-EYFP fusion protein expression, and DSC2b-pLPCX plasmids for fl-DSC2b-expression.

### Primary Antibodies

The clone 3B11 (anti-DSG2-3B11 [65]; Figure S10) directed against the extracellular domain of DSG2 was used for indirect immunofluorescence detection either with a microscope or by flow cytometry. To detect His-tagged proteins by flow cytometry, a FITC-conjugated anti-6x His tag® antibody (anti-HisFITC; ab1206, Abcam) was used. The affinity-isolated rabbit anti DSC2 (anti-DSC2; HPA01911, Sigma) and a rabbit polyclonal antibody to N-cadherin IgG (anti-NCad; ab18203, Abcam) were used for indirect immunofluorescence detection either with a microscope or by flow cytometry.

For Western blotting a murine IgG1 antibody against the extracellular domain of DSG2 clone 10G11 (anti-DSG2-10G11; BM5016, Acris Antibodies GmbH), a murine IgG1 antibody clone DG3.10 against the intracellular domain of DSG1+2 (anti-DSG1+2-DG3.10; BM370, Acris Antibodies GmbH) and a rabbit polyclonal antibody to PDI IgG (anti-PDI; ab31811, Abcam) as an endoplasmatic reticulum marker were used.

### Transfection of HT1080 cells

For all transfections, Lipofectamine™ 2000 (LFA; Life Technologies) was used according to the manufacturer’s protocol.

Transient transfections were performed with 400 ng fl-DSG2-pEYFP-N1 plasmid DNA and 7×10^4^ cells/0.8 cm^2^ in Lab-Tek™ chambered coverglasses (Thermo Scientific). Cells were cultivated for 24 h.

For stable transfection 800 ng DSG2-EC1-4-RGS-6xHis-pLPCX or DSC2b-pLPCX plasmid DNA was transfected into 2×10^5^ cells in a 24-well dish. Transfected cells were selected with medium containing puromycin (PAA). Single cell colonies were used for expression.

For DSG2 siRNA knock-down 2×10^5^ HT1080 cells were reversely transfected with 100 nM DSG2 Silencer® Select Pre-designed siRNA (ID s2773; Life Technologies). As a control LFA mock transfected cells and cells transfected with Silencer® Select Negative Control #1 siRNA (Life Technologies) were used. Knock-down efficiency was analysed 96 h after transfection by flow cytometry and Western blot. For Western blot cells were lysed by subcellular fractionation [66] and the membranous fraction was analysed.

### Purification of Recombinant Proteins

rECDs expressed in HT1080 and collected from cell culture supernatant were dialysed against native or denaturating (4 M urea) HisTrap binding buffer and purified by affinity chromatography on HisTrap™ HP Columns (GE Healthcare), according to the manufacturer’s instructions. Proteins were eluted with HisTrap native or denaturating elution buffer, respectively, and dialysed against a urea-free buffer.

The protein solutions were concentrated with Amicon® Ultra-4 Centrifugal Filter Units (10,000 MWCO, Millipore). Proteins were identified by Western blot and MALDI-PMF. To determine the purity of rECD, proteins were analysed by SDS-PAGE with subsequent Coomassie-R-250 staining. Coomassie-R-250 staining was quantified with AlphaView v.3.2.2.0 (ProteinSimple).

### MALDI-ISD

For MALDI-ISD at least 0.1 mg/ml (1.5 µM) of the undigested proteins in 10 mM HEPES, 150 mM NaCl, pH 7.5, were spotted on the MTP AnchorChip 800/384 TF and co-crystallised 1∶1 with a saturated solution of 1,5-diaminonaphthalene in 50% (v/v) acetonitrile, 0.1% (v/v) trifluoroacetic acid in H_2_O.

All spectra were acquired on an ultrafleXtreme with smartbeam-II™ solid state 1 kHz laser, FlashDetector™, and PAN™ broadband resolution with FlexControl software v.3.3 (BrukerDaltonics).

Data were Analysed with the Biotools Software v.3.2 (Bruker Daltonics).

### Circular Dichroism Spectroscopy (CD)

For CD spectroscopy, the purified rECDs were displayed against 10 mM Tris-HCl, pH 7.5, and measured at a concentration of 0.09–0.42 mg/ml with or without 5 mM CaCl_2_. The CD spectra were recorded on a J-810 spectrometer (Jasco) in a 1 mm quartz cell at 22°C, using a scanning rate of 50 nm min^−1^, a data pitch of 0.1 nm and three accumulations. All spectra were measured in the range of 350 to 190 nm and subjected to baseline correction. Deconvolution of the CD spectra was performed with DichroWeb [67]. For secondary structure estimation the average of all matching solutions obtained with CONTINLL based on the CONTIN algorithm [68], [69] and the CRYST175 reference database were used [70].

### Chemical Crosslinking

For crosslinking 0.8 µM rECD in 10 mM HEPES, pH 7.5, 150 mM NaCl, and 5 mM CaCl_2_ were incubated with BS^3^ (Sigma Aldrich) at a final concentration of 10 µM at 22°C for 60 min. The reaction was stopped by adding Tris-HCl, pH 7.5 to a final concentration of 90 mM. Negative controls were incubated under the same conditions but without BS^3^.

### Flow Cytometry Binding Assay

HT1080 cells were collected with enzyme-free PBS-based cell dissociation buffer (Life Technologies). 2.5×10^5^ cells were incubated either with primary antibody or with rECD for 30 min at 4°C in a final volume of 50 µl with 5 mM CaCl_2_. After washing with ice cold PBS bound primary antibody and rECD were incubated with a fluorescence-dye conjugated antibody for 30 min at 4°C in the dark. After washing with ice cold PBS cells were resuspended in PBS and analysed by flow cytometry with FACScan (Becton Dickinson). As a negative control cells only incubated with the fluorescence-dye conjugated antibody were used.

For flow cytometric analysis after knock-down, siRNA and mock transfected cells were used 96 h after transfection.

### Immunofluorescence of Transiently Transfected HT1080 Cells

Growth medium was substituted 24 h after transfection with microscopy buffer (150 mM NaCl, 20 mM HEPES, pH 7.4, 1 mM CaCl_2_, 5 mM KCl, 1 mM MgCl_2_, 0.19 (w/v) glucose, 0.19 (w/v) albumin (Fraktion V, Roth)) preheated to 37°C. Images were acquired on Eclipse TE2000-U (Nikon) with CCD-1300B (VDS Vosskühler GmbH) using Lucia G (Nikon GmbH). Image acquisition times for one filter were kept equal for all examined samples. LFA mock transfected cells were used as a negative control.

For indirect immunofluorescence of HT1080 cells, see Supporting Information.

### Statistical Analyses

Statistical analyses were performed with the unpaired student’s t-test, one-way ANOVA with Dunnett’s or Bonferroni’s posttest, or two-way ANOVA using Prism v5.01 (GraphPad Software, San Diego California USA). All indicated values are presented as means±standard error of the mean (SEM).

Detailed protocols can be obtained from the Supporting Information.

## Supporting Information

Figure S1 **Molar circular dichroism (Δε) of rECD-wt-nat (A) and rECD-wt-denat (B) presented against the wavelength.** rECD-wt-nat was purified under native conditions, rECD-wt-denat under denaturating conditions. Shown are the means (dark blue or red points)±SEMs (light blue or pink bars) for three independent measurements with (blue marks) or without (red/pink marks) CaCl_S_. The addition of 5 mM CaCl_2_ led to a significant increase of Δε in the range of 200–240 nm.
(TIF)

Figure S2 **Cleavage site sequence logos.** Shown are the specifity preferences of four PCs in each of the subsites P4 to P4‘ (1 = P4, 8 = P4‘) according to the MEROPS database [7], [8]. For explanations on how to interpret the cleavage site sequence logo compare Crooks et al. [8].
(TIF)

Figure S3 **Analysis of cadherin expression on HT1080 cells with immunofluorescence microscopy.** To verify the expression of cadherins on the human fibrosarcoma cell line HT1080 [9] unpermeabilised cells were labelled with anti-DSC2 (**A**), anti-DSG2-3B11 (**B**), and anti-NCad (**C**) antibodies and corresponding FITC- (**A+C**) or Cy^TM^3-conjugated (**B**) secondary antibodies, respectively. Negative controls performed with the secondary antibody only (not shown) did not show any specific fluorescence under the same conditions. Nuclear staining was performed with DAPI (blue). Immunofluoresecence microscopy revealed that DSG2, N-cadherin and in traces DSC2, the cadherins of the *area composita* in the human ID, are also expressed on HT1080. N-cadherin localises especially at the cell borders whereas DSC2 and DSG2 are scattered over the cell surface. Scale (red or green bar) = 10 µm.
(TIF)

Figure S4 **Flow cytometry analysis of HT1080.** Cells were brought into suspension with enzyme free cell dissociation buffer to avoid the cleavage of cadherins from the cell surface and incubated with rECD and the appropriate antibodies at 4°C to inhibit epitope endocytosis. For the discrimination between live/dead cell population cells were subsequently incubated with PI. Representative FL1/FL2 (**A**) dot plot shows one population (blue) with a low FL2 fluorescence intensity and two populations with high FL2 fluorescence intensity (pink, red circle). High FL2 fluorescence intensity corresponds to a PI uptake characteristic of dead cells. The dead cells in **A** correspond to the pink population in the representative SSC/FSC dot plot (**B**). The blue cell population in **B** corresponding to live cells was gated. Only the cells in the live cell gate (70–80% of all cells) were considered for the flow cytometry-based binding assay.
(TIF)

Figure S5 **Flow cytometric detection of DSG2 on HT1080.** Depletion of Ca^2+^ has a negative effect on the detection of DSG2 on HT1080. **A** HT1080 cells were incubated with (black line) or without (negative control, grey filled area) anti-DSG2-3B11. Bound antibody was detected with anti-msFITC. Shown are histograms of FITC fluorescence for DSG2 detection with 5 mM CaCl_2_ (1) or with 2 mM EGTA (2). **B** Column plots representing the ratio of DSG2 detection related to the negative control. Ratios are indicated as mean±SEM of 3 independent measurements. Fluorescence intensity ratio was significantly (p<0.0001) decreased from 1.57±0.01 for samples incubated in 5 mM CaCl_2_ (1) to 1.31±0.02 for samples incubated with 2 mM EGTA (2). Statistical analysis was performed with unpaired student’s t-test (GraphPad Prism 5.01).
(TIF)

Figure S6 **Detection of DSG2 on HT1080 after siRNA knock-down.** Treatment with DSG2-specific siRNA led to a reduction of DSG2 in HT1080. **A** Shown are representative histograms of FITC fluorescence for the detection of DSG2 with anti-DSG2-3B11+anti-msFITC on HT1080 in control cells (1, black line and 2, red line) and after DSG2-specific siRNA knock-down (3, green line). As negative control only anti-msFITC (grey filled area) was used. **B** Column plots represent the ratio of flow cytometric DSG2 detection (ratio_DSG2_; for calculation see formula S4) related to the negative control. Ratios_DSG2_ are indicated as mean±SEM of 3 independent knock down experiments. Statistical analysis was performed with one-way ANOVA with Bonferroni’s posttest (GraphPad Prism 5.01). Even control siRNA treatment significantly decreased surface DSG2 on HT1080 cells. After DSG2-specific siRNA knock-down surface DSG2 was undetectable by flow cytometry. **C** Western blot analysis of the membranous fraction (5 µg/lane) of HT1080 cells with anti-DSG1+2-DG3.10 (red box) and anti-PDI (grey box, ca. 60 kDa) as ER marker and loading control. Full-length DSG2 (ca. 165 kDa) and a cleavage fragment (ca. 105 kDa) were detectable in all three samples. DSG2 expression was obviously reduced in the lysate derived from DSG2 siRNA treated cells. Loading marker PDI showed that variability in protein loading could not account for the observed DSG2 decrease in the DSG2 siRNA treated sample. Due to high non-specific binding antibodies against the extracellular domain of DSG2 (anti-DSG2-3B11 and anti-DSG2-10G11) were not suitable for Western blot analysis of cell lysates.
(TIF)

Figure S7 **Flow cytometry-based assay for the binding of rECD-wt to HT1080 cells after siRNA knock-down. A** Shown are representative histogram plots of FITC-fluorescence for rECD-wt (0.8 µM) binding to HT1080. Bound rECD-wt was detected with anti-HisFITC. As negative control only anti-HisFITC (grey filled area) was used. **B** Column plots represent the ratio of rECD-wt-binding (ratio_rECD-bound_; calculation according to formula S3) to HT1080. Values represent the mean ratio±SEM of 3 independent knock-down experiments. Analysis was performed with one-way ANOVA and Bonferroni’s posttest (GraphPad Prism 5.01). Since rECDs were able to form homo-oligomers in solution (Figure 5C) and DSC2 for hetero-oligomer formation is only rarely present at the cell surface (Figure S3) we assumed that rECDs bind to DSG2 expressed by HT1080 cells. Nevertheless rECD-wt-binding was not influenced by DSG2 siRNA knock-down.
(TIF)

Figure S8 **DSC2b overexpression in HT1080 cells leads to increased surface expression of endogenous DSG2.** In wild type HT1080 cells neither overexpression of fl-DSG2 nor the expression of chimeric fl-DSG2-wt-EYFP was possible (data not shown). **A** Immunofluorescence analysis of DSC2b-HT1080 cells. To verify the expression of DSC2 unpermeabilised HT1080 cells stably transfected with DSC2b (DSC2b-HT1080) were labelled with anti-DSC2 (+) and anti-rbFITC as a secondary antibody (green). Nuclear staining was performed with DAPI (blue). Shown are representative immunofluorescence images for three immunolabelling experiments. The controls were treated with anti-rbFITC only (-). DSC2 expression was increased in DSC2b-HT1080 as compared to HT1080 (see also Figure S3); protein localised particularly at the cell borders. Scale (white bars) = 10 µm. **B** Flow cytometric analysis of DSG2 and DSC2 expression on HT1080 or DSC2b-HT1080 cells. Cells were treated with anti-DSG2-3B11+anti-msFITC and anti-DSC2+anti-rbFITC, respectively and analysed by flow cytometry. Column plots represent the ratio of DSG2 or DSC2 on HT1080 cells (ratio_desmosomal cadherin_; for calculation compare ratio_DSG2_, formula S4) as compared to the negative control (secondary antibody only). Values represent the mean ratio±SEM of 3 independent flow cytometry experiments; statistical analysis was performed with two-way ANOVA (GraphPad Prism 5.01). The analysis reveals that stable transfection of HT1080 cells with a human DSC2b construct (fl-DSC2b-pLPCX) and overexpression of DSC2b in DSC2b-HT1080 lead to an increased expression of endogenous DSG2 as compared to wild type HT1080. This is in agreement with the assumption that DSC-surface expression is necessary for DSG2-membrane transport [10]. Since DSC2b overexpression promotes DSG2 expression, DSC2b-HT1080 cells were used for fl-DSG2-EYFP expression.
(TIF)

Figure S9 **Protein alignment of murine N-cadherin (mNCad), murine E-cadherin (mECad) and human desmoglein-2 (hDSG2).** The Ca^2+^ binding motifs as annotated for murine N-cadherin (PDB ID 3Q2W) and murine E-cadherin (PDB ID 3Q2V) are highlighted in yellow [11]. The amino acids in hDSG2 corresponding to D154 and D187 are indicated in red and green, respectively. It is obvious that the ARVC-associated variations D154E and D187G concern conserved Ca^2+^-binding motifs. The multiple sequence alignment was performed with ClustalW. ‘*’ indicates positions which have a single, fully conserved residue, ‘:’ indicates that one of the ‘strong’ groups is fully conserved,‘.’ indicates that one of the ‘weaker’ groups is fully conserved.
(TIF)

Figure S10 **Detection of rECDs with anti-DSG2-3B11 by Western blot.** Although rECD-wt and all variants except rECD-R46Q were shown to lack the prodomain by MALDI-ISD (Figure 3) recombinantly expressed proteins were stained with anti-DSG2-3B11 by Western blot analysis. anti-DSG2-3B11 was claimed to be a DSG2-prodomain specific antibody, previously [12] but it seems to detect also DSG2 fragments lacking the prodomain. We used anti-DSG2-3B11 as a DSG2 extracellular domain specific antibody in our study. 1-6 = rECDs as labelled in Figure 1.
(TIF)

Table S1 **Oligonucleotides used for cloning and the corresponding sequences.** The bases used for the addition of a particular tag or for mutagenesis are written in capital letters.
(XLSX)

Methods S1 **Supporting Information: Material and Methods.**
(DOC)
