# Supplementary figures and images for: In Vitro Functional Analyses of Arrhythmogenic Right Ventricular Cardiomyopathy-Associated Desmoglein-2-Missense Variations
Source: PLoS One. 2012 Oct 10;7(10):e47097. doi: 10.1371/journal.pone.0047097 (PMC3468437; doi:10.1371/journal.pone.0047097)

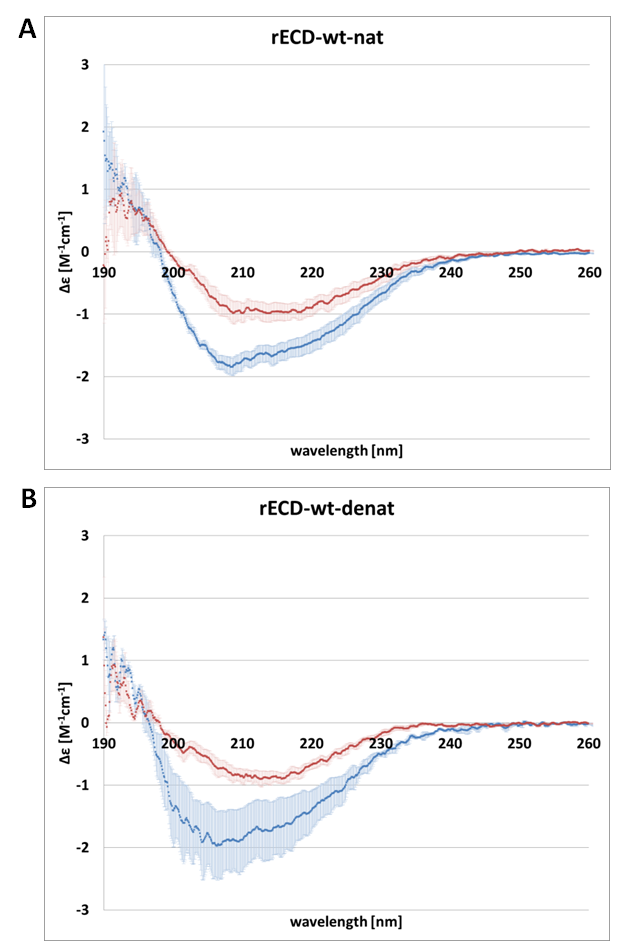

Supplement: Figure S1 — Molar circular dichroism (Δε) of rECD-wt-nat (A) and rECD-wt-denat (B) presented against the wavelength. rECD-wt-nat was purified under native conditions, rECD-wt-denat under denaturating conditions. Shown are the means (dark blue or red points)±SEMs (light blue or pink bars) for three independent measurements with (blue marks) or without (red/pink marks) CaClS. The addition of 5 mM CaCl2 led to a significant increase of Δε in the range of 200–240 nm. (TIF) [file pone.0047097.s001.tif]

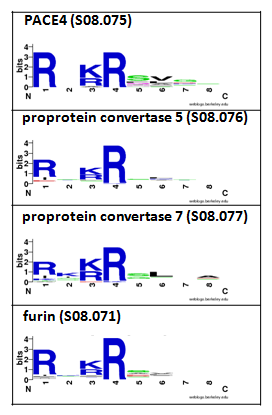

Supplement: Figure S2 — Cleavage site sequence logos. Shown are the specifity preferences of four PCs in each of the subsites P4 to P4‘ (1 = P4, 8 = P4‘) according to the MEROPS database [7], [8]. For explanations on how to interpret the cleavage site sequence logo compare Crooks et al. [8]. (TIF) [file pone.0047097.s002.tif]

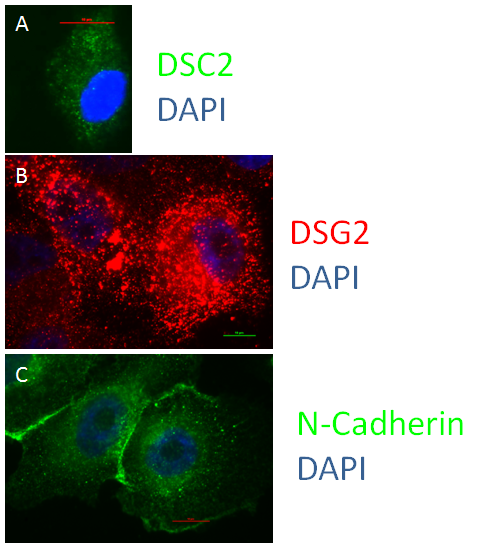

Supplement: Figure S3 — Analysis of cadherin expression on HT1080 cells with immunofluorescence microscopy. To verify the expression of cadherins on the human fibrosarcoma cell line HT1080 [9] unpermeabilised cells were labelled with anti-DSC2 (A), anti-DSG2-3B11 (B), and anti-NCad (C) antibodies and corresponding FITC- (A+C) or CyTM3-conjugated (B) secondary antibodies, respectively. Negative controls performed with the secondary antibody only (not shown) did not show any specific fluorescence under the same conditions. Nuclear staining was performed with DAPI (blue). Immunofluoresecence microscopy revealed that DSG2, N-cadherin and in traces DSC2, the cadherins of the area composita in the human ID, are also expressed on HT1080. N-cadherin localises especially at the cell borders whereas DSC2 and DSG2 are scattered over the cell surface. Scale (red or green bar) = 10 µm. (TIF) [file pone.0047097.s003.tif]

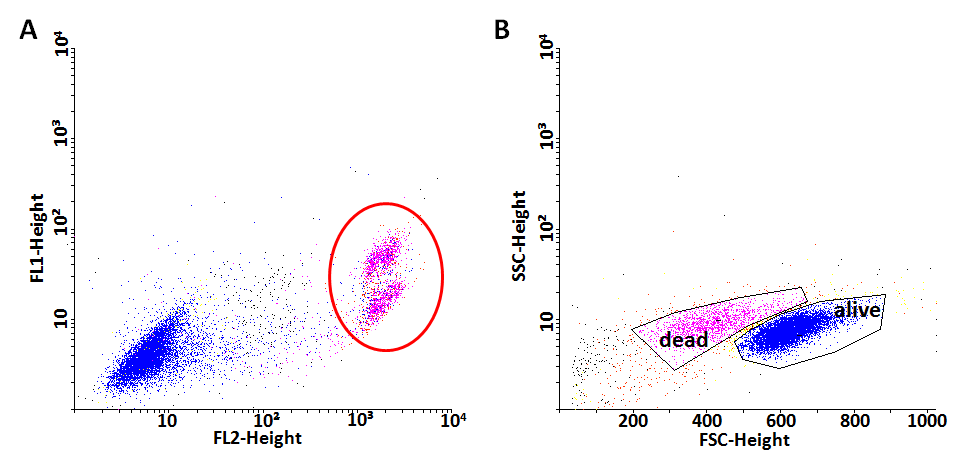

Supplement: Figure S4 — Flow cytometry analysis of HT1080. Cells were brought into suspension with enzyme free cell dissociation buffer to avoid the cleavage of cadherins from the cell surface and incubated with rECD and the appropriate antibodies at 4°C to inhibit epitope endocytosis. For the discrimination between live/dead cell population cells were subsequently incubated with PI. Representative FL1/FL2 (A) dot plot shows one population (blue) with a low FL2 fluorescence intensity and two populations with high FL2 fluorescence intensity (pink, red circle). High FL2 fluorescence intensity corresponds to a PI uptake characteristic of dead cells. The dead cells in A correspond to the pink population in the representative SSC/FSC dot plot (B). The blue cell population in B corresponding to live cells was gated. Only the cells in the live cell gate (70–80% of all cells) were considered for the flow cytometry-based binding assay. (TIF) [file pone.0047097.s004.tif]

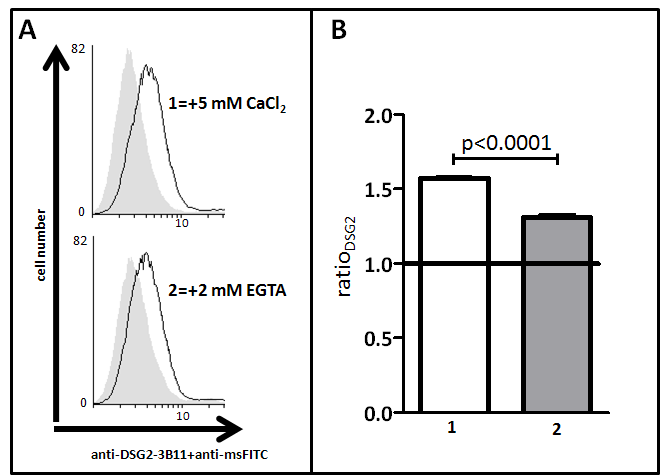

Supplement: Figure S5 — Flow cytometric detection of DSG2 on HT1080. Depletion of Ca2+ has a negative effect on the detection of DSG2 on HT1080. A HT1080 cells were incubated with (black line) or without (negative control, grey filled area) anti-DSG2-3B11. Bound antibody was detected with anti-msFITC. Shown are histograms of FITC fluorescence for DSG2 detection with 5 mM CaCl2 (1) or with 2 mM EGTA (2). B Column plots representing the ratio of DSG2 detection related to the negative control. Ratios are indicated as mean±SEM of 3 independent measurements. Fluorescence intensity ratio was significantly (p<0.0001) decreased from 1.57±0.01 for samples incubated in 5 mM CaCl2 (1) to 1.31±0.02 for samples incubated with 2 mM EGTA (2). Statistical analysis was performed with unpaired student’s t-test (GraphPad Prism 5.01). (TIF) [file pone.0047097.s005.tif]

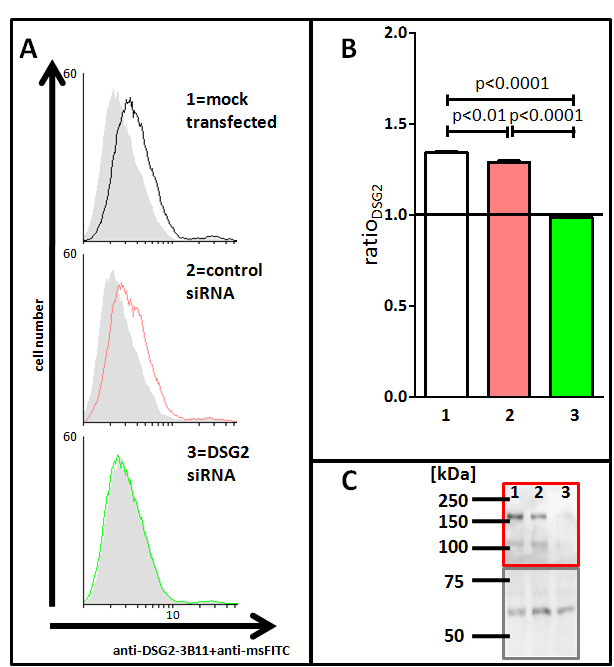

Supplement: Figure S6 — Detection of DSG2 on HT1080 after siRNA knock-down. Treatment with DSG2-specific siRNA led to a reduction of DSG2 in HT1080. A Shown are representative histograms of FITC fluorescence for the detection of DSG2 with anti-DSG2-3B11+anti-msFITC on HT1080 in control cells (1, black line and 2, red line) and after DSG2-specific siRNA knock-down (3, green line). As negative control only anti-msFITC (grey filled area) was used. B Column plots represent the ratio of flow cytometric DSG2 detection (ratioDSG2; for calculation see formula S4) related to the negative control. RatiosDSG2 are indicated as mean±SEM of 3 independent knock down experiments. Statistical analysis was performed with one-way ANOVA with Bonferroni’s posttest (GraphPad Prism 5.01). Even control siRNA treatment significantly decreased surface DSG2 on HT1080 cells. After DSG2-specific siRNA knock-down surface DSG2 was undetectable by flow cytometry. C Western blot analysis of the membranous fraction (5 µg/lane) of HT1080 cells with anti-DSG1+2-DG3.10 (red box) and anti-PDI (grey box, ca. 60 kDa) as ER marker and loading control. Full-length DSG2 (ca. 165 kDa) and a cleavage fragment (ca. 105 kDa) were detectable in all three samples. DSG2 expression was obviously reduced in the lysate derived from DSG2 siRNA treated cells. Loading marker PDI showed that variability in protein loading could not account for the observed DSG2 decrease in the DSG2 siRNA treated sample. Due to high non-specific binding antibodies against the extracellular domain of DSG2 (anti-DSG2-3B11 and anti-DSG2-10G11) were not suitable for Western blot analysis of cell lysates. (TIF) [file pone.0047097.s006.tif]

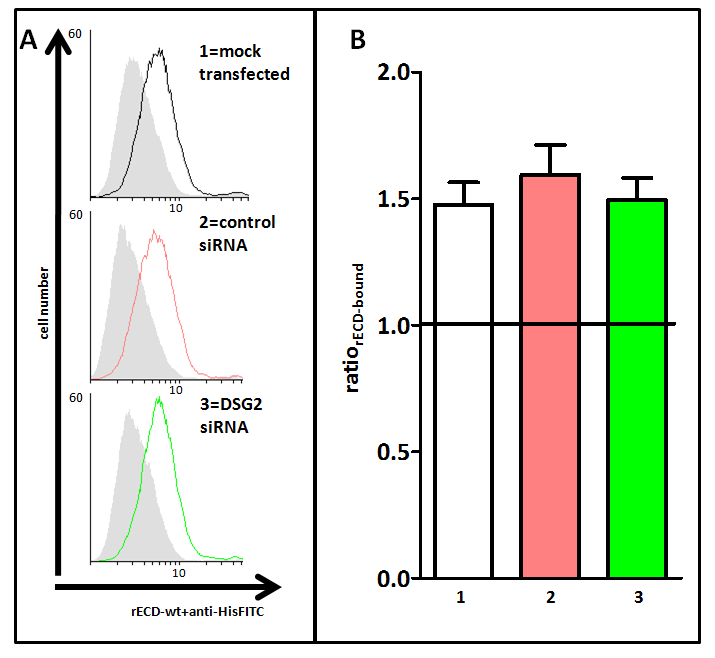

Supplement: Figure S7 — Flow cytometry-based assay for the binding of rECD-wt to HT1080 cells after siRNA knock-down. A Shown are representative histogram plots of FITC-fluorescence for rECD-wt (0.8 µM) binding to HT1080. Bound rECD-wt was detected with anti-HisFITC. As negative control only anti-HisFITC (grey filled area) was used. B Column plots represent the ratio of rECD-wt-binding (ratiorECD-bound; calculation according to formula S3) to HT1080. Values represent the mean ratio±SEM of 3 independent knock-down experiments. Analysis was performed with one-way ANOVA and Bonferroni’s posttest (GraphPad Prism 5.01). Since rECDs were able to form homo-oligomers in solution (Figure 5C) and DSC2 for hetero-oligomer formation is only rarely present at the cell surface (Figure S3) we assumed that rECDs bind to DSG2 expressed by HT1080 cells. Nevertheless rECD-wt-binding was not influenced by DSG2 siRNA knock-down. (TIF) [file pone.0047097.s007.tif]

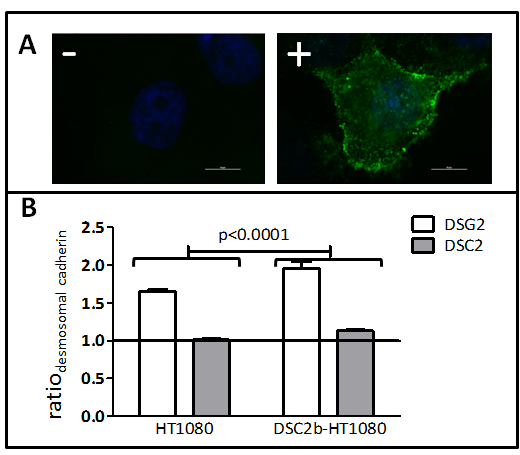

Supplement: Figure S8 — DSC2b overexpression in HT1080 cells leads to increased surface expression of endogenous DSG2. In wild type HT1080 cells neither overexpression of fl-DSG2 nor the expression of chimeric fl-DSG2-wt-EYFP was possible (data not shown). A Immunofluorescence analysis of DSC2b-HT1080 cells. To verify the expression of DSC2 unpermeabilised HT1080 cells stably transfected with DSC2b (DSC2b-HT1080) were labelled with anti-DSC2 (+) and anti-rbFITC as a secondary antibody (green). Nuclear staining was performed with DAPI (blue). Shown are representative immunofluorescence images for three immunolabelling experiments. The controls were treated with anti-rbFITC only (-). DSC2 expression was increased in DSC2b-HT1080 as compared to HT1080 (see also Figure S3); protein localised particularly at the cell borders. Scale (white bars) = 10 µm. B Flow cytometric analysis of DSG2 and DSC2 expression on HT1080 or DSC2b-HT1080 cells. Cells were treated with anti-DSG2-3B11+anti-msFITC and anti-DSC2+anti-rbFITC, respectively and analysed by flow cytometry. Column plots represent the ratio of DSG2 or DSC2 on HT1080 cells (ratiodesmosomal cadherin; for calculation compare ratioDSG2, formula S4) as compared to the negative control (secondary antibody only). Values represent the mean ratio±SEM of 3 independent flow cytometry experiments; statistical analysis was performed with two-way ANOVA (GraphPad Prism 5.01). The analysis reveals that stable transfection of HT1080 cells with a human DSC2b construct (fl-DSC2b-pLPCX) and overexpression of DSC2b in DSC2b-HT1080 lead to an increased expression of endogenous DSG2 as compared to wild type HT1080. This is in agreement with the assumption that DSC-surface expression is necessary for DSG2-membrane transport [10]. Since DSC2b overexpression promotes DSG2 expression, DSC2b-HT1080 cells were used for fl-DSG2-EYFP expression. (TIF) [file pone.0047097.s008.tif]

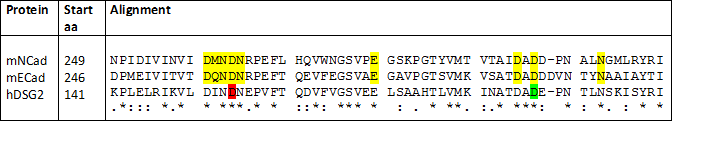

Supplement: Figure S9 — Protein alignment of murine N-cadherin (mNCad), murine E-cadherin (mECad) and human desmoglein-2 (hDSG2). The Ca2+ binding motifs as annotated for murine N-cadherin (PDB ID 3Q2W) and murine E-cadherin (PDB ID 3Q2V) are highlighted in yellow [11]. The amino acids in hDSG2 corresponding to D154 and D187 are indicated in red and green, respectively. It is obvious that the ARVC-associated variations D154E and D187G concern conserved Ca2+-binding motifs. The multiple sequence alignment was performed with ClustalW. ‘*’ indicates positions which have a single, fully conserved residue, ‘:’ indicates that one of the ‘strong’ groups is fully conserved,‘.’ indicates that one of the ‘weaker’ groups is fully conserved. (TIF) [file pone.0047097.s009.tif]

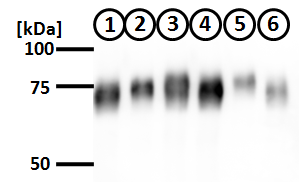

Supplement: Figure S10 — Detection of rECDs with anti-DSG2-3B11 by Western blot. Although rECD-wt and all variants except rECD-R46Q were shown to lack the prodomain by MALDI-ISD (Figure 3) recombinantly expressed proteins were stained with anti-DSG2-3B11 by Western blot analysis. anti-DSG2-3B11 was claimed to be a DSG2-prodomain specific antibody, previously [12] but it seems to detect also DSG2 fragments lacking the prodomain. We used anti-DSG2-3B11 as a DSG2 extracellular domain specific antibody in our study. 1-6 = rECDs as labelled in Figure 1. (TIF) [file pone.0047097.s010.tif]
