# Supplementary material for: In Vitro Functional Analyses of Arrhythmogenic Right Ventricular Cardiomyopathy-Associated Desmoglein-2-Missense Variations
Source: PLoS One. 2012 Oct 10;7(10):e47097. doi: 10.1371/journal.pone.0047097 (PMC3468437; doi:10.1371/journal.pone.0047097)
Supplement: Methods S1 — Supporting Information: Material and Methods. (DOC) [file pone.0047097.s012.doc]

**Supporting Information**

**Material and Methods**

**RNA isolation, cDNA synthesis, plasmid construction and mutagenesis**

DNA-free total RNA was isolated from human heart specimen using the RNeasy kit and RNase-free DNAse Set (Qiagen). RNA concentration was determined with a spectrophotometer at 260 nm. 100 ng of RNA were used as a template for cDNA synthesis with SuperScript®II Reverse Transcriptase (Life Technologies) with Random Primers (Invitrogen).

For cloning of DSG2-EC1-4-RGS-6xHis-pLPCX expression plasmids wild type (wt) cDNA and cDNA derived from the patient with the c.1174 G>A exchange were amplified with the FastStart High Fidelity PCR System (Roche). Using DSG2-EC1-4-RGS-6xHis-r and DSG2-f primers (for oligonucleotide sequence see Table S1) an RGS-His-Tag coding sequence and a NotI restriction site were added to the 3’-end and an XhoI restriction site to the 5’-end of positions 1-1509 of the DSG2 coding sequence. The PCR-products were first ligated into pCR®II-TOPO® (Life Technologies) and then subcloned into pLPCX (Clontech) using XhoI/NotI restriction sites. The sequence variations were inserted with the QuikChange® Lightning Site-Directed Mutagenesis Kit (Stratagene) into DSG2-EC1-4-wt-RGS-6xHis-pLPCX using the R46Q, D154E, D187G and K294E primers (Table S1).

For the generation of full-length DSG2 (fl-DSG2) expression plasmids cDNA was amplified with Phusion® High Fidelity DNA Polymerase (Thermo Scientific). DSG2-f and DSG2-r primers (Table S1) were used to add XhoI and BamHI restriction sites to the 5’-end and the 3’-end of the DSG2-coding sequence, respectively. After ligation into pCR®II-TOPO® the fragments were subcloned into pEYFP-N1 (Clontech) using XhoI and BamHI restriction sites. The mutations were inserted into fl-DSG2-wt-pEYFP with the QuikChange® Lightning Site-Directed Mutagenesis Kit (Stratagene) using the R46Q, D154E, D187G, K294E and V392I primers (Table S1).

For the generation of fl-DSC2b expression plasmids cDNA was amplified with Phusion® High Fidelity DNA Polymerase (Thermo Scientific). DSC2b-f and DSC2b-r primers (Table S1) were used to add XhoI and NotI restriction sites to the 5’-end and the 3’-end of the DSG2-coding sequence, respectively. After ligation into pCR®II-TOPO® the fragments were subcloned into pLPCX (Clontech) using XhoI and NotI restriction sites.

The accuracy of all cDNAs was verified by sequencing using the BigDye Terminator v3.1 cycle sequencing kit (Applied Biosystems^TM^ by Life Technologies^TM^) on an ABI 310 Genetic Analyzer (Applied Biosystems^TM^ by Life Technologies^TM^). Sequencing electropherograms were inspected manually using Chromas1.45 (Technelysium Pty Ltd). Sequences were compared to DSG2 coding sequence (NCBI Reference Sequence: NM_001943.3) using ClustalW online multiple sequence alignment software (http://www.genome.jp/tools/clustalw/).

**Secondary antibodies for primary antibody detection**

For immunofluorescence anti-DSG2-3B11 was detected with either Cy^TM^3-conjugated goat anti mouse IgG+IgM (anti-msCy3; 115-165-068, Dianova) or goat polyclonal to mouse IgG H&L, FITC-conjugated (anti-msFITC; ab6785, Abcam). anti-DSC2 and anti-NCad were detected with a goat polyclonal to rabbit IgG H&L, FITC-conjugated (anti-rbFITC; ab6717, Abcam).

For Western blotting the antibodies were detected either by a HRP-labeled goat anti mouse IgG (anti-msHRP; 554002, BD Biosciences) or by a donkey anti rabbit IgG, peroxidase-linked (anti-rbHRP, NA934, GE Healthcare).

**Cultivation of untransfected HT1080**

HT1080 cells were cultivated in high glucose DMEM (Gibco® by Life Technologies^TM^) supplemented with 10 % FCS Gold (PAA), Penicillin/Streptomycin and Amphotericin B (Biochrom AG) in a 5 % CO_2_ incubator at 37 °C.

**Selection of stably transfected rECD expressing subclones**

24 h after transfection of HT1080 with DSG2-EC1-4-RGS-6xHis-pLPCX the growth medium was substituted with selection medium (growth medium containing 600 ng/ml Puromycin (PAA)). A medium exchange was performed after 24 and 48 hours and the cells were grown till confluency. The confluent cells were subcloned and after 1-2 weeks single cell colonies were screened. The cell culture supernatants of each single cell colony were collected and analysed by Western blot. The colonies with a confirmed rECD expression were cultivated in selection medium and used as expression clones.

**Expression of recombinant proteins**

HT1080 cells stably transfected with plasmids coding for DSG2-EC1-4-RGS-6xHis-pLPCX were grown to ~90 % confluency on 145 mm cell culture dishes (Greiner CELLSTAR®). Selection medium was removed, the cells were washed with PBS (PAA) and supplemented with expression medium (selection medium without FCS). Cell culture supernatants were collected after 48 and 96 h, centrifuged at 4 °C and 1000xg for 5 minutes to remove cellular debris and supplemented with 4.72 % (w/v) (NH_4_)_2_SO_4_ (70 % saturation at 25°C ). Proteins were precipitated over night at 4 °C and centrifuged at 4 °C at 3900xg for 60 min. The pellets containing protein precipitates were resolved and dialysed against 20 mM NaH_2_PO_4_, 0.5 M NaCl, 3 mM DTT, pH 7.5 (HisTrap binding buffer) without urea (native conditions) or with 4 M urea (denaturating conditions). The purification was performed on HisTrap^TM^ HP Columns (GE Healthcare) according to the manufacturer’s instruction. The proteins were eluted with 20 mM NaH_2_PO_4_, 0.5 M NaCl, 0.5 M Imidazol, 3 mM DTT, pH 7.5 (HisTrap elution buffer) with or without 4 M urea. The elution fractions were analysed by SDS-PAGE with subsequent Coomassie-R-250 staining and Western blot, respectively. For further use fractions containing DSG2 (as detected by Western blot) and no other proteins (as shown by Coomassie-R-250 staining) were used. The selected elution fractions were stored at 4 °C and dialysed against a urea free buffer within one week.

**Calculation of rECD concentration**

The protein concentration was calculated by measuring the absorption at 280 nm and determining the exact protein concentration of purified rECD using the Beer-Lambert law with the molar absorption coefficient ε_280_ (M^-1^cm^-1^) according to (S1) .

**(S1)**

*n*W=number of tryptophan, *n*Y=number of tyrosine, *n*C=number of cystine residues. As for rECDs *n*W=3, *n*Y=17 and *n*C=2 ε_280_=42080 (M^-1^cm^-1^).

**Calculation of the molar circular dichroism (Δε)**

The molar circular dichroism (Δε) [M^-1^cm^-1^] was calculated using the formula S2


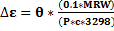
 **(S2)**

with θ=machine units [mdeg], MRW (mean residue weight)=molecular weight of the protein [Da]/number of amino acid residues-1), P=path length of the cuvette [cm] and c=concentration of the protein [mg/ml]. As MRW 70000/511 Da was used for the rECDs. P was 0.1 cm. For illustration of the results Δε was presented against the wavelength in nm. Only data in the far UV range (190-260 nm) were shown due to the low signal intensity in the near UV range (>260 nm) (Figure S1).

**Matrix and standards for MALDI-ISD; analysis of the spectra**

For data acquisition 5000 laser shots were accumulated from each spot by moving the sample holder randomly. For spectra acquisition RP_ISD.par method was used. Acquired spectra were automatically processed with Compass™ 1.3 software (Bruker Daltonics) using the SNAP algorithm for monoisotopic peak annotation. 1 mg/ml albumin solution (Fraktion V, Roth) in 10 mM HEPES, 150 mM NaCl, pH 7.5 was used for the internal calibration using the mass control list ”BSA_ISD mono.mcl. Buffer (10 mM HEPES, 150 mM NaCl, pH 7.5) was used as a negative control. Sequence analyses were carried out with BioTools™ 3.2 software (Bruker Daltonics). Peaks detected in the negative control were defined as background and neglected in the protein samples. ISD of proteins mainly produces c- and z-ions . For analysis the monoisotopic [M+H]^+^ masses of c-ions corresponding to *SS-Pro-ECD-*c-ions (DSG2-preproprotein), *Pro-ECD-*c-ions (DSG2-proprotein) and *ECD-*c-ions (mature DSG2) were calculated with the *MS/MS fragment ion calculator* from The Institute of Systems Biology at

http://db.systemsbiology.net:8080/proteomicsToolkit/FragIonServlet.html.

Fragment ion peaks obtained by MALDI-ISD were manually compared to the expected peaks calculated with the *MS/MS fragment ion calculator* using QuPE .

**Flow cytometry-based binding assay: data acquisition and analysis**

HT1080 prepared for flow cytometric analysis were examined by FACScan (Becton Dickinson) with a voltage of E-1, 150, 450 and 410 and an AmpGain of 8.51, 2.10, 1.00, 1.00 in Lin, Log, Log, Log mode for FSC, SSC, FL1 and FL2, respectively.

Incubation with rECD was performed in 10 mM HEPES, pH 7.5, 150 mM NaCl, incubation with antibodies in high glucose DMEM. 5 mM CaCl_2_ or 2 mM EGTA were added to rECDs or primary antibodies where indicated and incubated for 1 h at 22 °C before incubation with HT1080.

Data analysis was performed with Cyflogic^TM^ v.1.2.1 (CyFlo Ltd, Finland, http://www.cyflogic.com/index.php?link1=1). The geometric means (GeoMeans) of fluorescence intensity of cells in the live cell gate were used for statistical analysis. The ratio of specifically bound rECD (ratio_rECD-bound_) was determined by (S3).


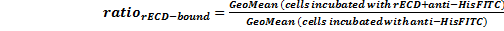
 **(S3)**

The ratio of specifically detected epitope (e.g. ratio_DSG2_ for DSG2) was calculated by (S4).


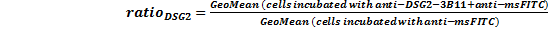
 **(S4)**

In the absence of specific binding the ratio was 1.

For the comparison of binding of the different rECDs HT1080 cells were treated with the same molar concentrations of rECDs in 5 mM CaCl_2_ and analysed by flow cytometry. For better comparability between rECD-wt- and rECD-variants-binding properties in each round of rECD-variant-binding the binding of rECD-wt was measured, too. In total 7 independent rounds of rECD-variant-binding with rECDs from at least 3 independent purifications were performed.

For analysis of the siRNA knock-down experiment cells treated for 96 h with DSG2-specific siRNA, unspecific siRNA and mock transfected cells were incubated with anti-DSG2-3B11 and detected with anti-msFITC by flow cytometry. As a negative control cells were incubated with anti-msFITC only.

**Characterisation of the live cell population for the flow cytometric assay**

To characterise the live cell population antibody treated cells were incubated with 12.5 ng/µl propidium iodide (PI, Beckman Coulter) for 15 min in the dark. As PI only diffuses through the ruptured membrane of dead cells these could be detected as a population with high fluorescence in the FL2 channel; cells with a low fluorescence in the FL2 channel were regarded as live cells. Cells in the FSC/SSC dot plot corresponding to the low FL2 fluorescence cells in the FL2/FL1 dot plot were gated. For the subsequent analysis only the cells in the live cell gate were used.

**Subcellular fractionation of HT1080 and determination of protein concentration with BCA assay**

For subcellular fractionation a protocol published previously was used with moderate modifications. Briefly, cells were washed with PBS and incubated with enzyme free PBS-based cell dissociation buffer (Gibco® by Life Technologies^TM^) for 5 min at 37 °C. Cells were rigorously resuspended in growth medium and centrifuged for 5 min at 100xg and 4 °C. Pellet was washed in ice-cold PBS, resuspended in 150 mM NaCl, 50 mM HEPES, pH 7.4, 100 µg/ml digitonin and incubated on ice for 10 min. Cells were centrifuged for 5 min at 2000xg and 4 °C, resuspended in 150 mM NaCl, 50 mM HEPES, pH 7.4, 1 % (v/v) Nonidet® P40 substitute (Sigma-Aldrich), incubated on ice for 30 min and centrifuged for 5 min at 2000xg and 4 °C. The supernatant representing the membranous fraction was collected.

Protein concentration of the membranous fraction was determined with the BCA assay . Briefly, protein solutions were supplied with a BCA/CuSO_4_ (50:1) solution and incubated at 37 °C for 30 min. Absorption of protein solutions was measured at 562 nm. Different concentrations of albumin were used for the creation of the standard curve.

**Indirect immunofluorescence of HT1080 cells**

Cells were spread on coverglasses coated with a 1:10 dilution of 0.2 % collagen R-solution (SERVA electrophoresis). After 24 h cells were fixed with Roti®-HistoFix 4 % (Roth) for 15 min at 22°C, washed with PBS and incubated with primary antibody in 1 % (w/v) albumin (Fraktion V, Roth)/PBS (PAA) solution for 30 min at 22°C. Subsequently PBS washed cells were treated with a fluorescent-dye conjugated secondary antibody for 30 min at 22°C in the dark. Finally cells were incubated with 1*10^-7^ % (w/v) DAPI-solution (Roth) for 5 min at 22°C in the dark. Cells were washed with ddH_2_O and mounted with Fluorescent Mounting Medium (DAKO). Cells only incubated with a fluorescent-dye conjugated secondary antibody were used as negative control.

Image acquisition was performed as described in the main manuscript.

**Protein analysis with SDS-PAGE, Western blot, Coomassie-R-250-Staining**

Protein samples were supplied 1:1 with 125 mM Tris, 4 % (w/v) SDS, 20 % (v/v) glycerol, 1 % (v/v) β-mercaptoethanol, xylenecyanole, pH 7.0 as loading buffer and separated by discontinuous SDS-PAGE (for gel preparation compare http://www.carlroth.com/media/_de-de/usage/3037.pdf with 30% acrylamid/bisacrylamid solution 29.1/0.9).

For Western blotting proteins were transferred on 0.45 µm nitrocellulose membranes (BioRad) using 48 mM Tris, 39 mM glycin, 0.0375 % (w/v) SDS, 20 % (v/v) methanol with the semi-dry method. For blocking 5 % skim milk solution in TTBS (0.1 % (v/v) Tween 20, 20 mM Tris, pH 7.5, 0.1 M NaCl) was used. Antibody incubation was performed in TTBS as indicated. For signal detection membranes were incubated with ChemiGlow West Chemiluminescence Substrate Kit (ProteinSimple) and chemoluminescence was detected with the FluorChem2 Imaging System (Biozym).

Coomassie-R-250 staining was performed with 0.1 % (w/v) Coomassie-R-250 in 40 % (v/v) ethanol, 10 % (v/v) acetic acid.

**References**

1. Green AA, Hughes WL (1955) Protein fractionation on the basis of solubility in aqueous solutions of salts and organic solvents. Methods in Enzymology 1: 67-90.

2. Pace CN, Vajdos F, Fee L, Grimsley G, Gray T (1995) How to measure and predict the molar absorption coefficient of a protein. Protein Sci 4: 2411-2423.

3. Demeure K, Quinton L, Gabelica V, De Pauw E (2007) Rational selection of the optimum MALDI matrix for top-down proteomics by in-source decay. Anal Chem 79: 8678-8685.

4. Albaum SP, Neuweger H, Franzel B, Lange S, Mertens D, et al. (2009) Qupe--a Rich Internet Application to take a step forward in the analysis of mass spectrometry-based quantitative proteomics experiments. Bioinformatics 25: 3128-3134.

5. Holden P, Horton WA (2009) Crude subcellular fractionation of cultured mammalian cell lines. BMC Res Notes 2: 243.

6. Smith PK, Krohn RI, Hermanson GT, Mallia AK, Gartner FH, et al. (1985) Measurement of protein using bicinchoninic acid. Anal Biochem 150: 76-85.

7. Rawlings ND, Barrett AJ, Bateman A (2010) MEROPS: the peptidase database. Nucleic Acids Res 38: D227-233.

8. Crooks GE, Hon G, Chandonia JM, Brenner SE (2004) WebLogo: a sequence logo generator. Genome Res 14: 1188-1190.

9. Rasheed S, Nelson-Rees WA, Toth EM, Arnstein P, Gardner MB (1974) Characterization of a newly derived human sarcoma cell line (HT-1080). Cancer 33: 1027-1033.

10. Burdett ID, Sullivan KH (2002) Desmosome assembly in MDCK cells: transport of precursors to the cell surface occurs by two phases of vesicular traffic and involves major changes in centrosome and Golgi location during a Ca(2+) shift. Exp Cell Res 276: 296-309.

11. Harrison OJ, Jin X, Hong S, Bahna F, Ahlsen G, et al. (2011) The extracellular architecture of adherens junctions revealed by crystal structures of type I cadherins. Structure 19: 244-256.

12. Keim SA, Johnson KR, Wheelock MJ, Wahl JK, 3rd (2008) Generation and characterization of monoclonal antibodies against the proregion of human desmoglein-2. Hybridoma (Larchmt) 27: 249-258.
